# Clinical application value of metagenomic next-generation sequencing in children with fever of unknown origin

**DOI:** 10.3389/fped.2026.1868060

**Published:** 2026-08-05

**Authors:** Shihua Liu, Shaoning Wang, Jian Li

**Affiliations:** Department of Infectious Diseases, Children's Hospital Affiliated to Shandong University (Jinan Children's Hospital), Jinan, Shandong Province, China

**Keywords:** children, fever of unknown origin, infectious diseases, metagenomic next-generation sequencing, pathogen detection

## Abstract

**Purpose:**

Infectious diseases constitute the predominant cause of fever of unknown origin (FUO). Conventional microbiological testing is limited by prolonged turnaround times, susceptibility to host/environmental interference, low detection sensitivity, and limited capacity to identify rare pathogens. Metagenomic next-generation sequencing (mNGS) enables parallel broad-spectrum screening for microbial agents. This study aimed to investigate the clinical utility of mNGS in children presenting with FUO, to generate descriptive observational data on pathogen detection and temporally associated anti-infective regimen adjustments.

**Methods:**

This retrospective single-center analysis enrolled 41 hospitalized children diagnosed with FUO who underwent mNGS testing at the Department of Infectious Diseases, Affiliated Children's Hospital of Shandong University, from June 2022 to July 2025. Initially, all patients underwent comprehensive routine systemic evaluations. For cases where fever persisted despite conventional testing and an infectious etiology was highly suspected, or where there was a poor therapeutic response to empirical anti-infective treatments, mNGS was subsequently performed. All specimens submitted for testing were sterile body fluids. Each sample was divided into two aliquots: one was subjected to conventional microbiological testing (including culture, smear microscopy, and PCR), while the other was cryopreserved for mNGS analysis. The performance of pathogen detection was compared between mNGS and conventional testing modalities using paired specimen data.

**Results:**

In this study, we analyzed 41 pediatric cases, which included three types of specimens: blood, cerebrospinal fluid (CSF), and tissue fluid (comprising deep pus, postoperative drainage fluid, subdural effusion, and aspirated fluid from the mass). mNGS identified 30 microbial isolates from 20 patients, which included bacteria, viruses, fungi, and mycoplasmas; of these, 17 isolates were ultimately confirmed as causative pathogens. No statistically significant differences in positivity rates were observed between mNGS and conventional assays, as indicated by paired 2 × 2 contingency tables (all *P* > 0.05).The present study also recorded changes to antimicrobial regimens that occurred after pathogen identification by mNGS testing, including adjuvant antiviral therapy for 4 patients, antimicrobial escalation for 6 patients, antimicrobial de-escalation for 2 patients, and comprehensive regimen modifications for an additional 5 patients.

**Conclusion:**

We analyzed a targeted pediatric FUO subgroup, and the overall pathogen detection positivity rate showed no statistical difference between mNGS and routine microbial testing. Accordingly, mNGS cannot currently replace standard workflows or routinely screen all FUO children. The two testing methods exhibited complementary pathogen detection spectra. mNGS may act as an auxiliary tool for complicated infectious cases with negative conventional test results. This study generates descriptive observational data on pathogen identification and temporally associated anti-infective regimen adjustments in a selected cohort of FUO children. Further prospective studies with larger sample sizes are required to validate these findings.

## 1 Introduction

Pediatric FUO is a prevalent, complex condition with atypical manifestations. Given rapid deterioration risks in children, timely etiologic diagnosis is indispensable. The absence of globally standardized diagnostic criteria and treatment guidelines for pediatric FUO impedes early identification and clinical intervention, and represents a major contributor to mortality among affected patients (1). The definition of FUO was first formalized by Petersdorf and Beeson in 1961 (2): fever persisting for a minimum of three weeks; recurrent febrile episodes exceeding 38.3 °C; and absence of a definitive diagnosis following one week of inpatient diagnostic evaluation. Integrating contemporary international and domestic literature with real-world clinical practice, FUO is stratified into classic FUO and FUO in special populations. The latter subgroup further encompasses hospital-acquired FUO and FUO associated with immunodeficiency (3–5). Classic FUO involves patients without underlying immunodeficiency presenting with sustained fever exceeding three weeks, at least three oral temperature readings above 38.3 °C (or diurnal temperature fluctuations >1.2 °C documented on a minimum of three occasions), and unresolved etiology following ≥7 days of systematic diagnostic workup. Systematic examinations should include routine blood, urine and stool (with occult blood) test, liver/renal function, serum electrolytes, inflammatory markers (C-reactive protein, procalcitonin and erythrocyte sedimentation rate), blood culture, chest computed tomography (CT), and abdominal imaging [ultrasound, CT/magnetic resonance imaging (MRI)] covering the liver, gallbladder, pancreas, spleen and kidneys (3–5). The most appropriate current definition of children's FUO provides for a temperature above 38.3 °C, at least once a day, for more than 8 days in a child evaluated on an outpatient basis by the pediatrician (with inconclusive history, physical examination, and first-level investigations) (6). Considering China's clinical context, the present study primarily enrolled patients fulfilling classic FUO diagnostic criteria, with case screening protocols refined in accordance with contemporary peer-reviewed literature (7).

FUO etiologies include infections, malignancies, non-infectious inflammatory diseases (NIIDs), and miscellaneous conditions. While the proportion of NIID-associated FUO has risen over recent decades, particularly within European cohorts, infectious agents remain the leading global cause (37%); malignancies constitute the third most prevalent etiology at 15% of cases (7). In Chinese cohorts, infections cause over 50% of cases (3, 8), followed by NIIDs (10 to 30%), malignancies (10 to 20%), and miscellaneous pathologies (5 to 15%) (3). Rapid, accurate pathogen identification thus constitutes a critical step in managing infectious FUO. Over recent years, diagnostic modalities have expanded beyond nonspecific laboratory markers (complete blood count, C-reactive protein, procalcitonin) to cross sectional imaging, immune profiling, nucleic acid testing, and invasive procedures (lumbar puncture, bone marrow aspiration, tissue biopsy) (3). Over the last 70 years, the proportion of FUO conditions has risen in industrialized countries with 7%–53% undiagnosed patients (7).

Metagenomic next-generation sequencing (mNGS) is extensively validated for etiologic diagnosis of infectious diseases, e.g., eschar-free scrub typhus (9), severe acute respiratory syndrome coronavirus 2 (10). mNGS performance exhibits reduced susceptibility to prior antimicrobial exposure relative to conventional culture-based assays (11). Prior investigations have validated the robust diagnostic efficacy of mNGS in pediatric infectious diseases and FUO cohorts. In pediatric cohorts, mNGS outperforms conventional method tests (CMTs) by improving viral detection in suspected bloodstream infections (12), diagnosing atypical cat scratch disease (13), and identifying Tanzanian acute febrile outbreak pathogens (14). mNGS also guides therapy for community-acquired pneumonia (15), pulmonary infections (16), mucormycosis (17), acute osteomyelitis (18), and unexplained encephalitis (19), while discovering novel pathogens like three uncharacterized viruses in Ugandan children (20).

The application of mNGS in pediatric populations warrants further descriptive investigation. Existing mNGS investigations predominantly focus on single-organ infections. Stratified pediatric FUO analyses, parallel sterile fluid assessments, and quantitative evidence for mNGS-associated antimicrobial regimen modifications remain scarce. Addressing these gaps, this single-center retrospective analysis reviewed mNGS-tested pediatric FUO patients at the Infectious Diseases Department of the Affiliated Children's Hospital of Shandong University. Paired specimen comparisons quantified pathogen detection differences between mNGS and conventional assays, and generated preliminary observational data regarding antimicrobial regimen adjustments following mNGS testing.

## 2 Study design and methods

### 2.1 Study population

This retrospective single-center clinical investigation enrolled 41 pediatric patients hospitalized at the Department of Infectious Diseases, Affiliated Children's Hospital of Shandong University, between June 2022 and July 2025. Eligible patients received mNGS testing and possessed complete inpatient medical records. Case inclusion adhered to classic FUO diagnostic criteria (3), with screening workflows refined per published clinical guidelines (7). Inclusion criteria: Age ≤ 18 years; hospitalization duration exceeding seven days with recurrent fever ≥38.3 °C, or sustained fever persisting for more than three weeks with a minimum of three oral temperature readings ≥38.3 °C (or diurnal temperature fluctuations >1.2 °C documented on ≥3 separate occasions). Exclusion criteria: incomplete electronic medical record documentation; immunocompromised patients (e.g., human immunodeficiency virus-positive individuals, solid organ transplant recipients); fever secondary to nosocomial infection developing ≥48 h post-admission. This study was approved by the Institutional Ethics Committee of the Affiliated Children's Hospital of Shandong University (Ethics Approval No. SDFE-JRB/T-2026004), De-identified inpatient data were analyzed, and the ethics committee approved a waiver of guardians' informed consent.

### 2.2 Research methods

A standardized tiered diagnostic procedure was implemented for all participants. All 41 patients first underwent standardized systemic diagnostic workups, including complete blood count, inflammatory biomarker quantification, cross-sectional imaging, and conventional pathogen culture/PCR. For patients with unresolved fever, the cause remains unclear following conventional testing, high clinical suspicion of an infectious etiology, or suboptimal therapeutic response to empirical anti-infective therapy, mNGS testing was subsequently ordered.

Demographic covariates (age, biological sex) and clinical metadata (medical history, comorbidities, prior antimicrobial exposure, and clinical outcomes) were extracted from inpatient electronic health records. All diagnostic specimens collected were sterile body fluids including peripheral blood, cerebrospinal fluid (CSF) and tissue fluids (deep pus, postoperative drainage fluid, subdural effusion, aspirated fluid of the mass) from the same patient. All tissue fluid samples were obtained via ultrasound- or CT-guided puncture. Specimens were collected in the priority order of CSF > tissue fluid > peripheral blood and sent for detection immediately. Delayed submission required standardized cryopreservation per institutional laboratory protocols with strict sterile operation throughout the whole process.

Each collected specimen was partitioned into two identical aliquots: one aliquot was submitted to the hospital clinical microbiology laboratory for conventional pathogen detection (culture, smear microscopy, PCR), while the second aliquot was blood at room temperature, CSF and tissue fluids were cryopreserved at −20 °C for batch mNGS analysis. Specimens were transported via a continuous cold-chain logistics to an external collaborating clinical laboratory, where mNGS was performed using the IDseq™ Ultra Pathogen Capture Metagenomic Sequencing Platform.

mNGS testing was performed using the IDseq™ Ultra pathogen capture metagenomic kit (Guangzhou Medical Device Record No. 20202085, etc.) matched with the VisionSeq1000 gene sequencer. Standardized experimental workflows were followed: total genomic DNA was extracted from each specimen, followed by nucleic acid purification and sequencing library construction prior to high-throughput sequencing. Two types of quality control samples, including negative extraction controls and blank amplification controls, were set in each batch of experiments simultaneously. All microbial read counts detected in these control samples were less than 10, eliminating exogenous nucleic acid contamination from the experimental workflow; uniform sequencing depth thresholds were enforced, ensuring a minimum of 20 million high-quality sequencing reads per specimen. Raw sequencing reads underwent quality control filtering to remove low-quality reads and host human genomic sequences. Remaining microbial-specific reads were aligned against MARS 3.0, an intelligent pathogen NGS management platform developed by MicroFuture Genomics.

This kit does not adopt a unified fixed cutoff value for microbial reads. At present, there is no globally consistent standard for interpreting pathogen reads via mNGS at home and abroad. All polymicrobial results were adjudicated by pediatric infectious disease physicians or microbiologists via tiered criteria to identify primary pathogens and rule out latent viruses/contaminants: Sequencing metrics (reads, RPM, genome coverage) were cross-referenced with batch negative controls to exclude low-abundance background signals; Specimen origin: low-level skin flora (e.g., Malassezia) from sterile fluids were deemed sampling contamination; Clinical evidence: dynamic inflammatory markers, imaging lesions and infectious symptoms; Confirmation via culture/PCR/serology where available. Microbes consistent with all the above evidence were defined as causative pathogens; organisms lacking active infection evidence were excluded. For mNGS-negative but culture/PCR-positive cases, clinical reassessment and repeat sequencing were arranged if indicated. This retrospective adjudication carries inherent interpretive bias. Statistical analyses were descriptive due to small subgroup sizes. Paired testing data from blood, tissue fluid and CSF were compared using the exact McNemar test; 95% confidence intervals (CIs) were calculated via the Clopper–Pearson exact method.

## 3 Results

### 3.1 Pediatric demographic and clinical data

Of the 41 enrolled children, 25 (61.0%) were male and 16 (39.0%) were female. Patients were stratified into pediatric age subgroups per national clinical classification standards: infant cohort (0–3 years, *n* = 12, 29.3%), preschool cohort (3–6 years, *n* = 9, 22.0%), school-age cohort (6–12 years, *n* = 17, 41.5%), and adolescent cohort (12–18 years, *n* = 3, 7.3%). All 41 patients exhibited febrile symptoms, with peak temperatures ranging from 38 °C to 41 °C. Isolated fever without localized organ-specific manifestations represented the most prevalent clinical presentation (20/41, 48.8%), followed by fever accompanied by cervical/axillary soft tissue masses (10/41, 24.4%), fever with neurological manifestations (seizures, cephalalgia, somnolence, ataxia; 6/41, 14.6%), and fever with respiratory symptoms (cough, chest tightness; 5/41, 12.2%).

Thirty-six patients (87.8%) received antimicrobial agents prior to hospital admission. At discharge, 37 children (90.2%) received a definitive diagnosis of an infectious disease, 3 (7.3%) were diagnosed with non-infectious inflammatory disorders, and 1 patient (2.4%) remained without a confirmed etiologic diagnosis. The 41 diagnostic specimens were distributed as CSF (17/41, 41.5%), tissue fluid (14/41, 34.1%), and peripheral blood (10/41, 24.4%). mNGS identified 17 validated causative pathogens across the cohort (17/41, 41.5%). Thirty-four patients (82.9%) achieved clinical cure or symptomatic improvement at hospital discharge. Full baseline and clinical metadata are summarized in Table 1.

**Table 1:** Summary of demographic and clinical data for 41 children with fever of unknown origin.

Category	Observation Item	Group/Outcome	Number of Cases	Constituent Ratio (%)
Baseline Information	Total subjects	—	41	100.0
	Gender	Male	25	61.0
		Female	16	39.0
	Age group (years old)	0 ≤ Age ＜ 3	12	29.3
		3 ≤ Age ＜ 6	9	22.0
		6 ≤ Age ＜ 12	17	41.5
		12 ≤ Age ≤ 18	3	7.3
	Underlying diseases	Yes	0	0.0
		No	41	100.0
	Chief complaint	Fever with respiratory symptoms	5	12.2
		Fever with neurological symptoms	6	14.6
		Fever with cervical/axillary masses	10	24.4
		Isolated fever without localized manifestations	20	48.8
	Peak temperature	< 38.3 ℃	6	14.6
		≥ 38.3 ℃	35	85.4
Diagnostic Information	Admission diagnosis	Fever of unknown origin	41	100.0
	Pre-admission antibiotic exposure	Yes	36	87.8
		No	1	2.4
		Unknown	4	9.8
	Discharge diagnosis	Infectious diseases	37	90.2
		Non-infectious inflammatory diseases	3	7.3
		Undetermined etiology	1	2.4
Core Research Indicators	Specimen type	Blood	10	24.4
		Tissue fluid（TF）	14	34.1
		Cerebrospinal fluid (CSF)	17	41.5
Conventional testing (culture, smear, PCR)	Blood	Positive	1	2.4
		Negative	9	22.0
	Tissue fluid	Positive	4	9.8
		Negative	10	24.4
	Cerebrospinal fluid	Positive	0	0.0
		Negative	17	41.5
mNGS Testing	Overall mNGS results	Positive (confirmed pathogenic microbes)	17	41.5
		Colonizing/contaminating microbes	13	31.7
		Negative	21	51.2
Treatment Outcomes	Regimen type	Antibacterial monotherapy	33	80.5
		Combined antibacterial and antiviral therapy	8	19.5
	Clinical prognosis	Cured/improved and discharged	34	82.9
		Discharged against medical advice	7	17.1

### 3.2 Pathogen detection by mNGS

Microbial sequences were detected in 20 of the 41 submitted specimens, with multiple co-infecting pathogens identified within individual samples and counted separately, yielding a total of 30 distinct microbial isolates. Tissue fluid specimens yielded the highest proportion of detected isolates (15/30, 50.0%), followed by peripheral blood (10/30, 33.3%) and CSF (5/30, 16.7%). Full specimen-matched pathogen distribution and isolate counts are presented in Table 2.

**Table 2:** Detection results of 30 pathogen by mNGS.

Specimen Source (Total strains in source)	Pathogen Species	Number of pathogen (Total *n* = 30)	Constituent Ratio within Specimen Type (%)
Tissue fluid (*n* = 15)	*Legionella pneumophila*	2	13.3
	*Staphylococcus aureus*	2	13.3
	*Streptococcus pyogenes*	2	13.3
	Human herpesvirus 4 (EBV)	3	20.0
	Human herpesvirus 5 (CMV)	1	6.7
	Torque teno virus (TTV)	1	6.7
	*Mycobacterium abscessus*	1	6.7
	*Streptococcus pneumoniae*	1	6.7
	*Prevotella oris*/*Prevotella loescheii*	1	6.7
	*Mycoplasma pneumoniae*	1	6.7
Blood (*n* = 10)	Torque teno virus (TTV)	3	30.0
	Human polyomavirus (BK virus)	1	10.0
	Human herpesvirus 1 (HSV-1)	1	10.0
	Human herpesvirus 4 (EBV)	2	20.0
	Human herpesvirus 5 (CMV)	1	10.0
	*Brucella* spp.	1	10.0
	*Streptococcus pneumoniae*	1	10.0
Cerebrospinal fluid (CSF) (*n* = 5)	Human herpesvirus 4 (EBV)	2	40.0
	Torque teno virus (TTV)	1	20.0
	*Brucella* spp.	1	20.0
	*Malassezia* spp.	1	20.0

Percentages were calculated per specimen type. Multiple pathogens identified in a single patient were counted independently.

**Figure 1:**
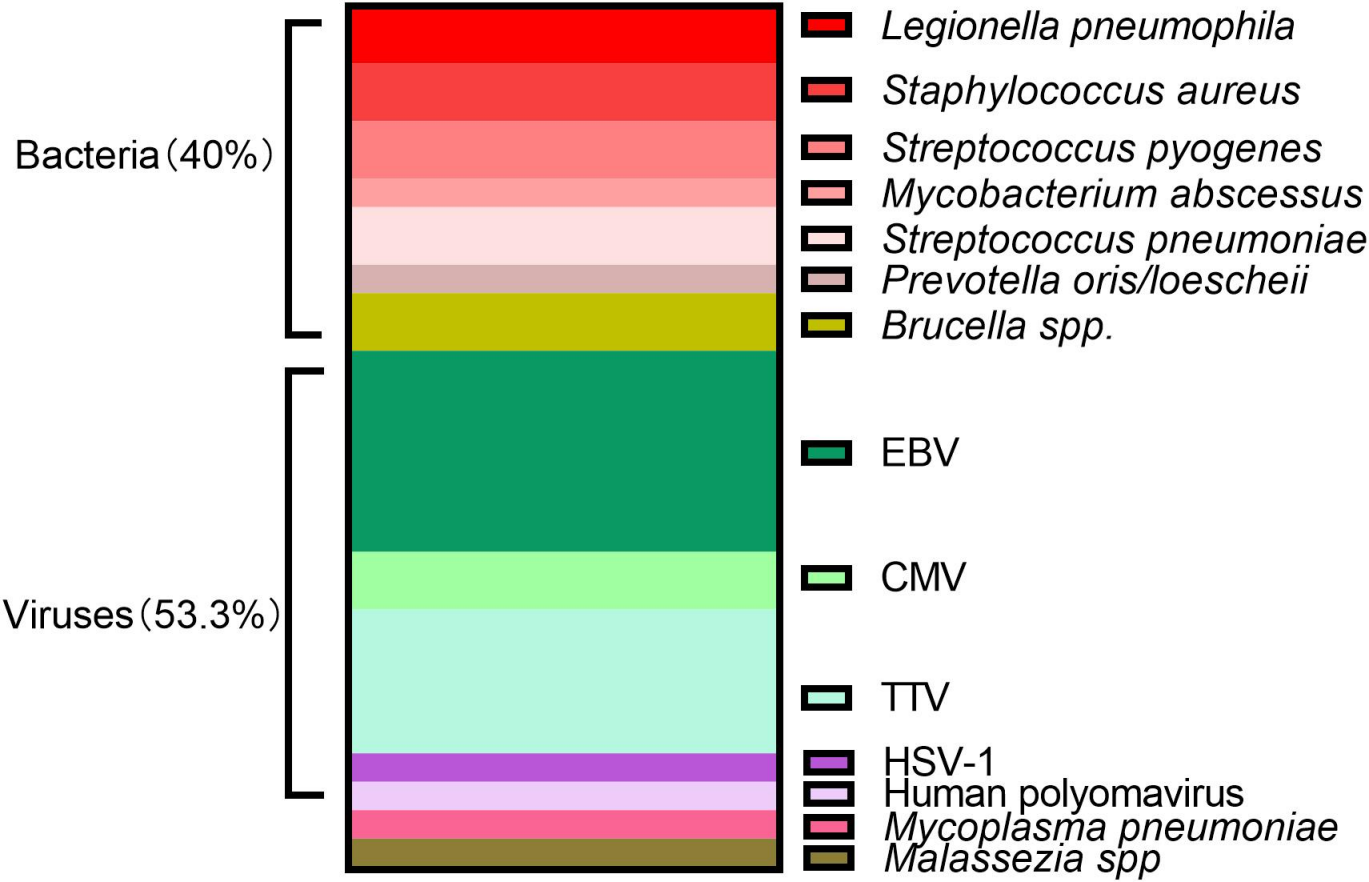
Detected pathogens via mNGS and Corresponding Strain Counts (30 Microbial Isolates in Total).

**Figure 2:**
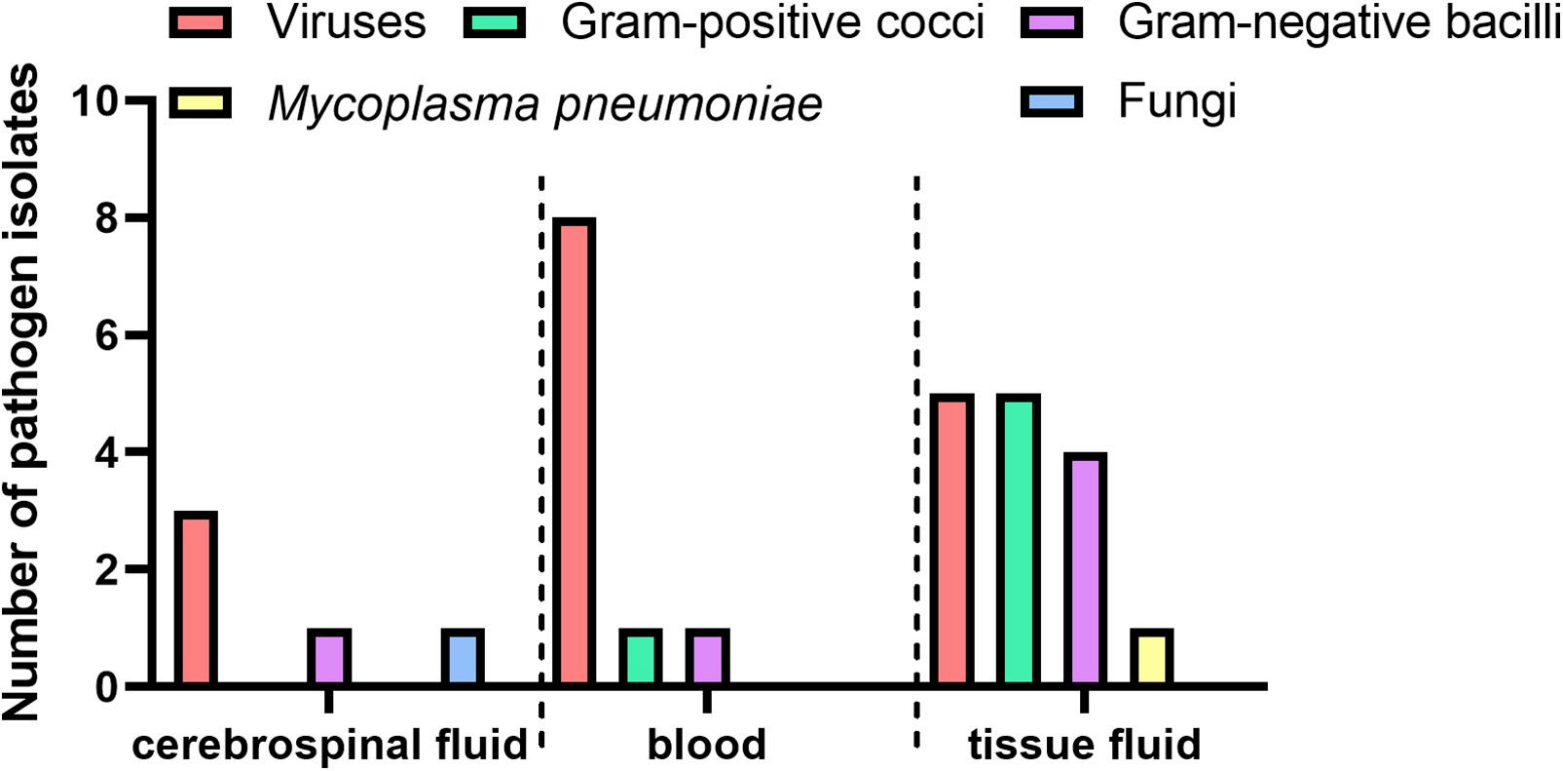
Distribution of microbial pathogens detected by mNGS across three specimen types (Total 30 Isolates).

Fourteen distinct microbial species were recovered, spanning bacteria, viruses, fungi, and mycoplasmas. Bacterial isolates accounted for 12 of the 30 total strains (40.0%), encompassing seven species, including four Gram-negative bacilli and three Gram-positive cocci. Viral isolates constituted the predominant pathogen class at 16 strains (53.3%), all classified as DNA viruses. Single isolates of Mycoplasma pneumoniae (1/30, 3.3%) and Malassezia spp. (1/30, 3.3%) were additionally recovered. The relative proportional distribution of all detected pathogens is visualized in Figure 1, while stratified pathogen distribution across blood, tissue fluid, and CSF matrices is illustrated in Figure 2.

### 3.3 Comparison of mNGS with conventional pathogen detection methods

Paired comparative performance metrics for mNGS and conventional testing across the three sterile body fluid matrices are summarized in Table 3. No statistically significant differences in detection positivity rates were identified between mNGS and conventional assays for any specimen matrix (all *P* > 0.05). The wide span of CIs in each group reflects imprecise point estimates secondary to small cohort sizes. Seventeen microorganisms detected by mNGS were clinically confirmed as causative pathogens after comprehensive evaluation. The overall turnaround time (TAT) of mNGS was approximately 24 h, compared with 48–72 h for conventional culture.

**Table 3:** Comparison of mNGS and conventional detection methods.

Specimen Type	mNGS+/ Conventional + (cases)	mNGS+/ Conventional− (cases)	mNGS−/Conventional + (cases)	mNGS−/Conventional− (cases)	mNGS positive rate (95%CI)	Conventional assay positive rate (95%CI)	*P* value for paired comparison (Exact McNemar test)
Blood	0	3	1	6	30.0%（6.67%∼65.25%）	10.0%（0.25%∼44.50%）	0.065
Tissue fluid	4	7	0	3	78.6%（49.20%∼95.34%）	28.6%（8.39%∼58.10%）	0.051
Cerebrospinal fluid (CSF)	0	3	0	14	17.6%（3.78%∼43.43%）	0.0%（0.00%∼19.49%）	0.249

All specimens were tested in parallel with mNGS and conventional assays.The four columns represent dual positive, mNGS-only positive, conventional-only positive, and dual negative cases respectively. Detection rates and 95% CIs were calculated via the Clopper–Pearson exact method. Pairwise comparisons were performed using the exact McNemar test. Discordant paired cases are listed below to assess the stability of marginal *P* values close to 0.05: blood (3 mNGS-only positive, 1 conventional-only positive); tissue fluid (7 mNGS-only positive, 0 conventional-only positive); cerebrospinal fluid (3 mNGS-only positive, 0 conventional-only positive). Small sample sizes lead to limited statistical power, and all *P* values > 0.05 indicate no significant difference in detection positivity rates between the two assays. the results are preliminary. We accept further independent statistical review if requested by the editorial team or reviewers.

The present study also recorded changes to antimicrobial regimens that occurred after pathogen identification by mNGS testing, including adjuvant antiviral therapy for 4 participants, antimicrobial escalation for 6 participants, antimicrobial de-escalation for 2 participants, and comprehensive multi-agent regimen modifications for 5 participants. Patients discharged against medical advice were followed up within half a year and recovered completely (Table 4 and Supplementary Table S4).

**Table 4:** Cases with temporally associated anti-infective regimen modifications following mNGS pathogen detection.

Case No.	Intervention Category	Specimen Type	Detected Pathogen	Read Count	Therapeutic Intervention	Clinical Outcome
1	Antiviral therapy added	Blood	Human herpesvirus 4 (EBV)	998	Acyclovir	Cured
2	—	Tissue fluid (subdural effusion)	Human herpesvirus 4 (EBV)	98	Acyclovir	Discharged against medical advice
3	—	Cerebrospinal fluid (CSF)	Human herpesvirus 4 (EBV)	534	Acyclovir	Cured
4	—	Cerebrospinal fluid (CSF)	Human herpesvirus 4 (EBV)	91	Acyclovir	Discharged against medical advice
5	Antibiotic escalation	Blood	*Streptococcus pneumoniae*	7,041	Linezolid	Cured
6	—	Tissue fluid（Ultrasound-guided deep pus aspiration）	*Staphylococcus aureus*	2,991	Linezolid	Cured
7	—	Tissue fluid（Ultrasound-guided deep pus aspiration）	*Staphylococcus aureus*	143,263	Linezolid	Improved
8	—	Tissue fluid（Ultrasound-guided fine-needle aspiration of mass）	*Streptococcus pyogenes*	78,532	Linezolid	Cured
9	—	Tissue fluid（Ultrasound-guided fine-needle aspiration of mass）	*Streptococcus pneumoniae*	1,554	Linezolid	Cured
10	—	Tissue fluid（Ultrasound-guided deep pus aspiration）	*Streptococcus pyogenes*	1,360	Linezolid	Improved
11	Regimen optimization	Blood	*Brucella spp.*	104	Doxycycline + Rifampicin	Discharged against medical advice
12	—	Cerebrospinal fluid (CSF)	*Brucella spp.*	480	Doxycycline + Rifampicin	Discharged against medical advice
13	—	Tissue fluid（Pleural effusion drainage）	*Mycoplasma pneumoniae*	631,854	Levofloxacin	Cured
14	—	Tissue fluid（Ultrasound-guided fine-needle aspiration of mass）	*Legionella pneumophila*	23,255	Azithromycin	Improved
15	—	Tissue fluid（Ultrasound-guided fine-needle aspiration of mass）	*Legionella pneumophila*	3,224	Azithromycin	Improved
16	Antibiotic de-escalation	Tissue fluid（Ultrasound-guided fine-needle aspiration of mass）	*Mycobacterium abscessus*	82	Azithromycin	Discharged against medical advice
17	—	Tissue fluid（CT-guided drainage for bilateral intracranial abscesses with subdural empyem	*Prevotella oris/Prevotella loescheii*	71	Metronidazole + Ceftriaxone	Improved

This table only summarizes cases where anti-infective regimens were adjusted after mNGS results were available. Given the retrospective single-center design without a matched control group, temporal correlation does not establish a causal link between mNGS testing and clinical improvement or therapeutic efficacy. No quantitative comparison of clinical outcomes (fever resolution, inflammatory marker recovery, length of hospitalization) between mNGS-modified and empirical therapy groups was performed in this study.

The remaining 13 mNGS detected microorganisms included five Torque teno virus (TTV) specimens (6 to 41 reads) establishing lifelong latency without organ pathogenicity. Human herpesvirus 5 (CMV) was identified in tissue fluid (deep abscess fluid) with a read count of 5, while peripheral blood yielded 10 matching reads; both samples exhibited negligible read abundances. Peripheral blood recovered 13 BK polyomavirus (BKV) reads. One peripheral blood specimen recovered 114 human herpesvirus 1 (HSV-1) reads; despite moderate absolute read counts, the absence of cutaneous herpes lesions, central nervous system herpetic encephalitis. Tissue fluid (aspirated fluid of the mass) specimens recovered minimal Epstein–Barr virus (EBV) read counts of 8 and 9, with peripheral blood yielding 12 matching reads; with low-level read counts. CSF recovered 4 *Malassezia spp*. reads; this fungal taxon constitutes the resident cutaneous flora, and minimal sequence recovery within sterile CSF. Evaluating these low-abundance signals alongside negative clinical, serological, and auxiliary evidence tends to suggest they represent latent nucleic acid release or specimen contamination without clinical pathogenic significance.

In addition, Streptococcus was isolated from blood culture after 72 h of incubation in one patient, while no pathogen was identified via mNGS. This organism was comprehensively judged as the causative pathogen, and the patient recovered and was discharged after adjusted treatment.

## 4 Discussion

Infectious diseases are the primary cause of fever of FUO in children, accounting for 19% to 69% of cases globally (6, 21). This single-center retrospective study exclusively included children with a high pre-test probability of infectious etiology, specifically those who exhibited negative results from conventional testing or inadequate therapeutic responses to empirical antimicrobial agents prior to mNGS testing. This approach introduces a selection bias. Furthermore, significant assay costs limited the enrollment to only 41 patients. Consequently, the proportion of confirmed infectious diseases (37/41, 90.2%) pertains solely to this selected subgroup and cannot be generalized to all pediatric FUO patients. Additionally, the small sample size further restricts the generalizability of the findings.

mNGS enables the simultaneous detection of a wide range of pathogens (22). Despite universal empirical antibacterial therapy, mNGS yielded a positive rate (20/41, 48.8%), recovering 14 distinct species across a total of 30 isolates, with DNA viruses identified as the predominant pathogen class. These findings are consistent with a retrospective analysis of pediatric FUO conducted at a single center (23). DNA viral genomes undergo direct sequencing following extraction, while RNA viruses necessitate additional reverse transcription to complementary DNA prior to library construction. This added step introduces technical complexity, which may reduce the sensitivity of RNA viral detection (24, 25). Furthermore, this suggests that mNGS holds significant diagnostic value for patients with a history of antibiotic exposure or those suffering from polymicrobial infections (11). Given false negative risks, illustrated by one culture-positive but mNGS-negative Streptococcus case, mNGS cannot completely replace conventional testing; rather, the two methods exhibit complementary pathogen spectra. Seventeen microbial isolates detected via mNGS were validated as definitive causative pathogens within the present cohort. Paired comparison demonstrated no statistically significant differences in positive detection rates between mNGS and conventional assays across blood, cerebrospinal fluid and tissue fluid specimens (all *P* > 0.05). This absence of statistical separation is primarily driven by insufficient sample sizes within individual specimen subgroups, a phenomenon previously documented across peer-reviewed mNGS diagnostic investigations (26). The median mNGS turnaround time across the cohort was approximately 24 h, vs. 48–72 h required for conventional microbial culture. This temporal difference in reporting speed was observed among children with complex severe infectious presentations.

For central nervous system fluid specimens such as cerebrospinal fluid and subdural effusion, detection of EBV with moderate read counts enables effective differentiation from low-load latent EBV in peripheral blood, which in this cohort was associated with the initiation of antiviral therapy. For severe infections involving *methicillin-resistant Staphylococcus aureus, Streptococcus pyogenes, Streptococcus pneumoniae*; rare intracellular bacteria, including *Brucella* spp. and *Legionella pneumophila*; anaerobes such as *Prevotella* spp, and slow-growing mycobacteria represented by *Mycobacterium abscessus*, this testing platform can detect pathogens undetectable by traditional culture methods, even in patients with prior antibiotic exposure. In this study, TTV, low-load CMV, HSV-1, BKV, and skin-derived *Malassezia* were all detected with trace read counts. No corresponding organ injury, serological or imaging evidence supporting pathogenicity was observed. These findings are considered to represent latent trace nucleic acids or background specimen contamination without definitive pathogenic evidence. Prudence is required for clinical interpretation, and these microbes should not be directly defined as colonizers. We documented multiple anti-infective regimen modifications that occurred temporally after mNGS pathogen identification. These observational treatment changes represent the primary descriptive outcome of this analysis, independent of statistical comparisons of pathogen detection rates between the two assays, and constitute the key observational output of the present cohort analysis.

This study has several limitations. As a single-center retrospective analysis with a limited sample size, it suffers from selection bias and restricted generalizability to only the described pediatric FUO subgroup. Furthermore, the limited inclusion criteria and missing serial inflammatory data, along with the absence of an mNGS-free control cohort, hinder our ability to evaluate the causal effects of mNGS on cure rates, hospital stays, antimicrobial exposure, and prognosis. Moreover, mNGS has well-documented clinical limitations (27): there is currently no universal standardized framework for the interpretation of mNGS sequencing outputs. Distinguishing pathogenic microbes from low-abundance background signals heavily relies on individual clinician expertise, which can lead to interpretive bias in this study. The technology does not generate antimicrobial susceptibility profiles, thereby limiting direct guidance for targeted therapy. Additionally, prohibitive assay costs, restricted detection performance for RNA viral species, the absence of large-cohort standardized validation data, and the lack of universal sequencing and quality control protocols further complicate the situation. Simultaneously capturing true pathogens and contaminants can lead to diagnostic confusion (28, 29). Further measures to address these technological and procedural limitations include optimizing sequencing workflows with RNA virus reverse transcription, unifying cutoffs and interpretation standards to reduce inter-clinician discrepancies, and establishing tiered testing protocols for different sterile fluids to define optimal mNGS indications.

In summary, no statistically significant intergroup differences in overall pathogen detection positivity rates were identified between mNGS and conventional microbiological testing modalities in this pediatric FUO subgroup. This real-world retrospective study reflects clinical mNGS use in pediatric FUO subgroups. Our documented regimen changes following mNGS testing merely represent descriptive observations from this selected cohort of children with FUO, rather than evidence confirming clinical therapeutic benefits. Future large, adequately powered multicenter prospective controlled trials are therefore required to eliminate selection bias, delineate the full pathogen spectrum, and quantify any measurable clinical benefits associated with mNGS application.

## Data Availability

The datasets presented in this article are not readily available because the raw genetic and genomic sequencing data cannot be publicly deposited in open repositories due to ethical restrictions and privacy protection requirements for pediatric participants, as approved by the institutional ethics committee. De-identified aggregated data are available from the corresponding author upon reasonable request with a signed confidentiality agreement.

## Supplementary material

Supplementary Table S4 Cases with temporally associated anti-infective regimen modifications following mNGS pathogen detection
