# Supplementary material for: Clinical application value of metagenomic next-generation sequencing in children with fever of unknown origin
Source: Front Pediatr. 2026 Aug 5;14:1868060. doi: 10.3389/fped.2026.1868060 (PMC13485945; doi:10.3389/fped.2026.1868060)
Supplement: Supplementary file 1 [file Table1.docx]

**Table S4.** **Cases with temporally associated anti-infective regimen modifications following mNGS pathogen detection**

| Case No. | Pre-adm Tx | Initial in-hosp Tx | WBC (×10⁹/L) | CRP（mg/L） | PCT（ng/ml） | mNGS TAT (h) | Norm Temp (d) | Norm Inflam (d) | Final Diagnosis |
| --- | --- | --- | --- | --- | --- | --- | --- | --- | --- |
| 1 | ceftizoxime | Cefotaxime, Meropenem | 21 | 98.69 | 0.89 | 24 | 3 | 15 | Sepsis, EBV infection |
| 2 | Mezlocillin-sulbactam, Reduning Injection | Ceftriaxone, ceftizoxime, meropenem | 17.78 | 11.45 | 0.207 | 24 | 3 | 9 | Intracranial infection, EBV infection |
| 3 | Ceftriaxone, ceftizoxime, azithromycin | Ceftizoxime, meropenem, vancomycin | 24.32 | 67.45 | 0.27 | 24 | 1 | 6 | Intracranial infection, sepsis, EBV infection |
| 4 | Azithromycin, intravenous immunoglobulin | Cefotaxime, meropenem | 15.55 | 11.33 | 0.05 | 24 | 2 | 7 | Viral encephalitis, EBV infection |
| 5 | Ceftizoxime | Cefotaxime, meropenem | 21.06 | 224.33 | 0.8 | 24 | 3 | 16 | Sepsis, otitis media |
| 6 | Clindamycin, cefoperazone-sulbactam | Cefoperazone-sulbactam | 10.73 | 24.65 | 3.04 | 24 | 10 | 9 | Soft tissue infection with abscess formation, MRSA infection |
| 7 | Ceftriaxone, meropenem | Cefoperazone-sulbactam | 7.49 | 5.77 | 0.07 | 24 | 1 | 11 | Soft tissue infection, Staphylococcus aureus infection |
| 8 | Mezlocillin-sulbactam | Mezlocillin-sulbactam, ceftriaxone | 12.1 | 31.93 | 0.04 | 24 | 2 | 2 | Cervical lymphadenitis complicated with Streptococcus pyogenes infection |
| 9 | Penicillins | Ceftriaxone, mezlocillin-sulbactam | 4.12 | <0.5 | 0.03 | 24 | 2 | 4 | Soft tissue infection, Streptococcus pneumoniae infection |
| 10 | Mezlocillin, cefoperazone-sulbactam | Ceftriaxone, mezlocillin-sulbactam, meropenem | 52.29 | 48.15 | 0.27 | 24 | 2 | 9 | Soft tissue infection |
| 11 | Cephalosporins | Ceftriaxone | 8.07 | 0.39 | 0.04 | 24 | 3 | 14 | Intracranial infection (neurobrucellosis) |
| 12 | Cephalosporins | Ceftriaxone | 5.92 | <0.449 | 0.04 | 24 | 3 | 14 | ntracranial infection (neurobrucellosis) |
| 13 | Cefoperazone-sulbactam, azithromycin, Reduning Injection | Ceftazidime, voriconazole | 18.6 | 0.81 | 0.16 | 24 | 2 | 11 | Lobar pneumonia, atelectasis, pleural effusion, Mycoplasma infection |
| 14 | Cefoperazone-sulbactam | Cefoperazone-sulbactam | 13.31 | 40.39 | 0.05 | 24 | 2 | 5 | Suppurative cervical lymphadenitis, Legionella pneumophila infection |
| 15 | Cephalosporins | Cefoperazone-sulbactam | 13 | 30 | 0.05 | 24 | 2 | 5 | Suppurative cervical lymphadenitis, Legionella pneumophila infection |
| 16 | Mezlocillin, cefoperazone-sulbactam | Cefoperazone-sulbactam, linezolid | 13.12 | 11.77 | 0.03 | 24 | 3 | No follow-up review | Soft tissue infection, Mycobacterium abscessus infection, lymphadenitis |
| 17 | Piperacillin | Meropenem, vancomycin | 27.36 | 32.53 | 0.23 | 24 | 4 | 4 | Intracranial infection |

Notes: Pre-adm Tx, pre-admission treatment; Initial in-hosp Tx, initial treatment after admission; WBC, white blood cell count; CRP, C-reactive protein; PCT, procalcitonin; TAT, turnaround time (the duration from specimen receipt to mNGS report release); Norm Temp, days for body temperature to return to normal after therapeutic adjustment; Norm Inflam, days for inflammatory markers to return to normal after therapeutic adjustment.
